# Supplementary material for: The Effects of Cyanobacterial Bloom Extracts on the Biomass, Chl-a, MC and Other Oligopeptides Contents in a Natural Planktothrix agardhii Population
Source: Int J Environ Res Public Health. 2020 Apr 22;17(8):2881. doi: 10.3390/ijerph17082881 (PMC7215471; doi:10.3390/ijerph17082881)
Supplement: Supplementary file 1 [file ijerph-17-02881-s001.zip › Table S1.docx]

**Table S1.** Oligopeptide composition of two different extracts of *P. agardhii*-dominated scum samples, the control *P. agardhii* and *P. agardhii* exposed for 7 days to the highest concentrations of the extracts. AER – aeruginosin, AERD – aeruginosamide, AP – anabaenopeptin, CPL – cyanopeptolin, MRG – microginin, MC – microcystin, PLP – planktopeptin, x and x/136 – two different classes of unidentified oligopeptides

| m/z [M+H]^+^ | Class | Extracts | | *P. agardhii* control | *P. agardhii* exposed to extract Pa-A | *P. agardhii* exposed to extract Pa-B |
| --- | --- | --- | --- | --- | --- | --- |
|  |  | Pa-A | Pb-B |  |  |  |
| 521 | [Asp^3^,MeSer^7^] MC-RR |  |  |  | + |  |
| 503 | x |  | + |  |  |  |
| 512 | [Asp^3^]MC-RR | + | + | + | + | + |
| 519 | MC-RR |  | + |  | + |  |
| 535 | AER |  | + |  |  |  |
| 561 | AERD |  | + |  |  | + |
| 564 | x |  | + |  |  |  |
| 573 | AER |  | + |  |  |  |
| 575 | AER | + | + |  |  |  |
| 576 | AER |  | + |  |  |  |
| 577 | AER |  | + |  |  |  |
| 579 | AER |  | + | + |  |  |
| 583 | AER |  |  | + | + | + |
| 585 | AER |  | + |  |  |  |
| 593 | AER |  | + |  |  |  |
| 599 | AER | + |  |  |  |  |
| 601 | AER | + |  |  |  |  |
| 603 | AER | + | + |  |  |  |
| 617 | AER |  | + |  |  | + |
| 627 | AER |  |  |  | + |  |
| 629 | MRG |  | + |  |  |  |
| 635 | AER |  | + |  | + | + |
| 637 | AER | + | + |  | + | + |
| 639 | AER | + |  | + |  |  |
| 650 | MRG |  |  |  | + |  |
| 659 | x | + |  |  |  |  |
| 679 | AER | + |  |  |  |  |
| 681 | AER | + |  |  |  |  |
| 685 | x | + | + |  |  |  |
| 691 | AER | + | + | + | + | + |
| 715 | AER | + | + | + | + | + |
| 718 | AER | + |  |  |  |  |
| 718 | AP | + |  |  |  |  |
| 725 | AER |  | + | + |  |  |
| 730 | MRG |  | + |  |  |  |
| 731 | AER | + | + |  |  |  |
| 740 | MRG |  | + |  |  |  |
| 749 | AER | + | + | + | + | + |
| 752 | AER | + | + |  |  |  |
| 755 | AER |  | + |  |  |  |
| 758 | AER |  |  | + |  |  |
| 759 | AER |  |  |  | + | + |
| 784 | x |  | + |  |  |  |
| m/z [M+H]^+^ | Class | Extracts | | *P. agardhii* control | *P. agardhii* exposed to extract Pa-A | *P. agardhii* exposed to extract Pa-B |
|  |  | Pa-A | Pb-B |  |  |  |
| 791 | AER |  | + |  |  |  |
| 792 | MRG |  | + |  |  |  |
| 801 | PLP | + | + | + |  |  |
| 828 | AP |  |  | + |  |  |
| 837 | AP | + |  | + | + | + |
| 839 | AP |  | + |  |  |  |
| 844 | AP | + |  | + | + | + |
| 847 | x |  | + |  |  |  |
| 849 | CPL | + |  | + | + |  |
| 851 | AP | + |  | + | + | + |
| 858 | AP | + | + |  |  |  |
| 859 | AP |  | + |  |  |  |
| 863 | CPL |  |  |  | + |  |
| 865 | CPL | + |  | + | + | + |
| 872 | AP |  | + |  |  |  |
| 878 | CPL |  | + |  |  |  |
| 879 | CPL | + |  | + | + | + |
| 881 | CPL |  |  |  | + |  |
| 885 | x/136 |  |  | + |  |  |
| 891 | x/136 |  |  | + |  |  |
| 893 | AP | + |  |  |  |  |
| 895 | AP | + |  |  |  |  |
| 897 | CPL |  | + |  |  |  |
| 904 | CPL |  | + |  |  |  |
| 909 | AP | + |  |  |  |  |
| 912 | CPL | + |  |  |  |  |
| 913 | CPL |  | + |  |  |  |
| 916 | CPL |  |  | + | + | + |
| 923 | AP | + |  |  | + | + |
| 927 | CPL | + |  |  |  |  |
| 929 | x/136 | + |  | + |  | + |
| 930 | CPL | + |  | + | + | + |
| 948 | CPL |  | + |  |  |  |
| 953 | x/136 | + | + | + |  | + |
| 959 | x/136 | + |  | + | + | + |
| 962 | x/136 |  | + |  |  |  |
| 976 | CPL |  | + |  |  |  |
| 977 | CPL |  | + |  |  |  |
| 978 | CPL | + |  |  |  |  |
| 981 | dmMC-LR | + | + | + | + | + |
| 986 | MC-LF |  | + |  |  |  |
| 992 | CPL | + |  |  |  |  |
| 995 | MC-LR | + | + |  |  |  |
| 1003 | CPL |  | + |  |  |  |
| 1006 | CPL | + |  |  |  |  |
| 1013 | x/136 | + |  |  |  |  |
| 1015 | x |  | + |  |  |  |
| 1021 | x | + |  | + |  |  |
| m/z [M+H]^+^ | Class | Extracts | | *P. agardhii* control | *P. agardhii* exposed to extract Pa-A | *P. agardhii* exposed to extract Pa-B |
|  |  | Pa-A | Pb-B |  |  |  |
| 1024 | [Asp^3^, Dhb^7^] MC-RR |  | + | + |  |  |
| 1032 | CPL |  |  | + |  | + |
| 1040 | CPL |  | + |  |  |  |
| 1042 | CPL | + |  |  |  |  |
| 1043 | CPL |  | + |  |  |  |
| 1045 | [Asp^3^]MC-HtyrR | + |  |  |  |  |
| 1058 | CPL | + |  |  |  |  |
| 1081 | x/136 | + |  |  |  |  |
| 1099 | x/136 | + |  |  |  |  |
| 1121 | x/136 | + |  |  |  |  |
| 1137 | x | + |  | + |  |  |
| 1161 | x/136 |  | + |  |  |  |
| Total | 103 | 50 | 55 | 29 | 25 | 23 |
