# Supplementary material for: The Effects of Cyanobacterial Bloom Extracts on the Biomass, Chl-a, MC and Other Oligopeptides Contents in a Natural Planktothrix agardhii Population
Source: Int J Environ Res Public Health. 2020 Apr 22;17(8):2881. doi: 10.3390/ijerph17082881 (PMC7215471; doi:10.3390/ijerph17082881)
Supplement: Supplementary file 1 [file ijerph-17-02881-s001.zip › Table S2.docx]

**Table S2.** Contribution of the biomass (%) of particular cyanobacterial taxa in the total biomass of phytoplankton after the 7-day exposure to extracts Pa-A and Pa-B originally containing different concentrations of MCs. Both extracts dilutions had similar concentration of Chl-a. Data are expressed as means ± SE, n=3.

|  | |  | **Chl-a concentrations in the treatments with the extract**  **Pa-A (mg l^-1^)** | | | |  | **Chl-a concentrations in the treatments with the extract**  **Pa-B (mg l^-1^)** | | | | |
| --- | --- | --- | --- | --- | --- | --- | --- | --- | --- | --- | --- | --- |
| **Taxa** | **Control** | | **0.32** | **0.64** | **1.28** | **2.56** | **Control** | | **0.33** | **0.66** | **1.32** | **2.64** |
| *Aphanizomenon gracile* | | 0.78  ±0.11 | 0.14  ±0.06 | 0.02  ±0.02 | 0.03  ±0.01 | 0.06  ±0.03 | 0.70  ±0.14 | 0.04  ±0.02 | | 0.01  ±0.01 | 0.15  ±0.14 | 0.23  ±0.15 |
| *Aphanocapsasa* spp. | | <0.01 | <0.01 |  | <0.01 |  | <0.01 | <0.01 | |  |  |  |
| *Aphanothece* sp. | | <0.01 | <0.01 | <0.01 | 1.50  ±0.21 | 2.25  ±0.27 | <0.01 | <0.01 | | 0.79  ±0.52 | 0.78  ±0.50 | 1.08  ±0.15 |
| *Chroococcus* spp. | | 0.03  ±0.03 |  |  | <0.01 |  | <0.01 | <0.01 | |  |  |  |
| *Cuspidothrix issatschenkoi* | | 0.10  ± 0.04 |  |  | 0.05  ± 0.05 | <0.01 | 0.13  ± 0.07 |  | | <0.01 | <0.01 |  |
| *Limnothrix redekei* | | <0.01 |  |  |  | <0.01 |  |  | |  |  |  |
| *Microcystis aeruginosa* | | <0.01 |  | <0.01 |  |  | <0.01 |  | |  |  |  |
| *Phormidium* sp. | |  |  |  |  | 0.01 |  |  | |  |  |  |
| *Planktolyngbya limnetica* | | 0.15  ±0.05 | 0.02  ±0.002 | 0.12  ±0.10 | 0.15  ±0.05 | 0.08  ±0.03 | 0.22  ±0.03 | 0.02  ±0.004 | | 0.03  ±0.01 | 0.14  ±0.02 | 0.03  ±0.02 |
| *Planktolyngbya* sp. | | 0.01  ±0.01 |  |  |  | 0.01  ±0.01 | 0.03  ±0.001 |  | | 0.01  ±0.01 | 0.01  ±0.01 |  |
| ***Planktothrix agardhii*** | | **97.77 ±0.38** | **99.28 ±0.22** | **99.44 ±0.32** | **97.61 ±0.42** | **97.01 ±0.37** | **97.71 ± 0.13** | **99.03 ±0.07** | | **98.79 ±0.45** | **98.26 ±0.12** | **97.53 ±0.17** |
| *Pseudanabaena* sp. | | 0.04 ±0.02 | 0.02 ±0.01 | 0.04 ±0.02 | 0.11 ±0.004 | 0.03 ±0.01 | 0.02 ±0.004 | 0.24 ±0.14 | | 0.06 ±0.03 | 0.06 ±0.03 | 0.05 ±0.02 |
| Total | | 98.87  ±0.47 | 99.45 ±0.27 | 99.62  ± 0.22 | 99.45 ±0.25 | 99.45 ±0.29 | 98.82  ± 0.04 | 99.34 ±0.21 | | 99.68 ±0.07 | 99.41  ± 0.07 | 98.92 ±0.12 |
